# Intensive Care Unit-Based Medical Education for Medical Students: A Scoping Review

**DOI:** 10.7759/cureus.88570

**Published:** 2025-07-23

**Authors:** Joseph L Kim, Yohan Kim

**Affiliations:** 1 Emergency Medicine, University of Wisconsin School of Medicine and Public Health, Madison, USA; 2 Neurocritical Care, University of Texas Southwestern Medical Center, Dallas, USA; 3 Neurology, University of Texas Health Science Center at San Antonio, San Antonio, USA

**Keywords:** clinical education, critical care education, graduate medical education, icu education, intensive care unit, medical education, medical student education, scoping review

## Abstract

As medical students and graduates are increasingly exposed and expected to recognize critically ill patients, the role of the intensive care unit (ICU) training in graduate medical education has drawn growing attention. This scoping review maps the current landscape of ICU-based education for medical students, focusing on the importance of early ICU exposure, teaching methods employed, learner outcomes, and gaps in literature. Peer-reviewed articles published between 2015 to 2025 were identified through searches from PubMed, Scopus, and Google Scholar using terms such as "medical education ICU" and "ICU training medical students". Titles and abstracts were screened, followed by full-text reviews. Twenty-four articles met inclusion criteria and were analyzed thematically. The findings suggest that ICU training improves knowledge, clinical skills, and self-confidence among medical students, especially when programs incorporate active learning strategies such as simulation, case-based teaching, and bedside participation. However, access to ICU rotations remains inconsistent, and many students worldwide report limited exposure. Gaps identified include a lack of standardized curricula, limited outcome-based research, and de-emphasis on non-technical skills like communication and ethics. While ICU education offers clear benefits, widespread integration into medical curricula remains uneven. Future efforts should prioritize curriculum development, faculty support, and equitable access to ensure all graduates are better prepared for acute care responsibilities.

## Introduction and background

Critical care medicine has become an increasingly important component of medical training in recent years. Foundational training in the intensive care unit (ICU) is now recognized as integral to preparing new physicians for the acute care of the critically ill. Yet many medical school graduates worldwide enter residency underprepared to manage critical patients [1]. A key reason is the inconsistent inclusion of ICU rotations or curricula in medical education. In fact, only a minority of medical schools require a dedicated ICU rotation, meaning many students graduate without any ICU experience [2]. This gap can leave new doctors feeling unready to recognize and treat life-threatening conditions. Early exposure to critical care during medical school has documented benefits, including reduced anxiety, greater confidence, and improved knowledge when caring for sick patients. It may even spark increased interest in critical care as a career [3]. Given the rising complexity of hospitalized patients and the expectation that all graduates can promptly identify and stabilize critically ill individuals, there is a clear need to assess how ICU-based education should be incorporated into early medical training.

This scoping review explores the current landscape of ICU-based medical education for medical students globally. We focus on the educational importance of ICU exposure, the teaching methods utilized in ICU settings, the reported outcomes of ICU education, and the gaps seen in literature (such as geographic disparities and hospital resources). By synthesizing findings from the past 10 years of peer-reviewed literature (2015-2025), we aim to map what is known and identify areas for improvement in preparing future physicians to care for critically ill patients. Additionally, two articles from pre-2015 were included to provide historical context and highlight how ICU medical education has evolved over time. Together, this review provides a comprehensive overview that can guide future research and curriculum development to enhance medical student training in critical care environments.

## Review

Methodology

Review Question

The objective of this scoping review is to examine how the intensive care unit is structured and evaluated for medical students in medical education. Specifically, this review focuses on the following review questions: 1) What types of educational interventions and instructional methods are used to teach medical students in the ICU? 2) What are the reported educational outcomes and perceived benefits of ICU-based training for medical students? and 3) What gaps, barriers, or inconsistencies exist that influence the delivery of ICU-based education for medical students?

Study Design

This scoping review was conducted using the Joanna Briggs Institute (JBI) Scoping Review methodology [4]. The Preferred Reporting Items for Systematic Reviews and Meta-Analyses extension for Scoping Reviews (PRISMA-ScR) checklist was used to report this review article [5].

Eligibility Criteria

The population-concept-context framework, as outlined by the JBI, was utilized as a guideline for the inclusion criteria for this scoping review. The articles included were published in peer-reviewed journals between January 2015 and May 2025. Additionally, two articles from 2001 and 2007 were included to provide historical context and serve as benchmarks in the development of ICU medical education. The population focuses majorly on medical students and excludes attending physicians, physician assistant students, and nurses. Although resident and fellow physicians were not the main focus, some articles included these groups to highlight key points relevant to the topic. The context of this scoping review included intensive care units (all types of ICUs) and excluded wards and non-ICU departments. The articles reported had qualitative or quantitative educational outcomes that focused on educational experiences, curricula, or teaching interventions in any ICU settings. Given the scoping nature of the review, formal quality appraisal was not performed; instead, a narrative overview of the evidence is presented.

Information Sources

The databases utilized for this scoping review included PubMed, Scopus, Google Scholar, and Web of Science.

Search Strategy

The search strategy included keywords such as "medical student", "medical education", "intensive care unit", "ICU", "critical care", "simulation ICU training", "critical care curriculum", "medical education", "education", "curriculum", and "teaching" (Appendix 1).

Study of Evidence Selection

**Figure 1:**
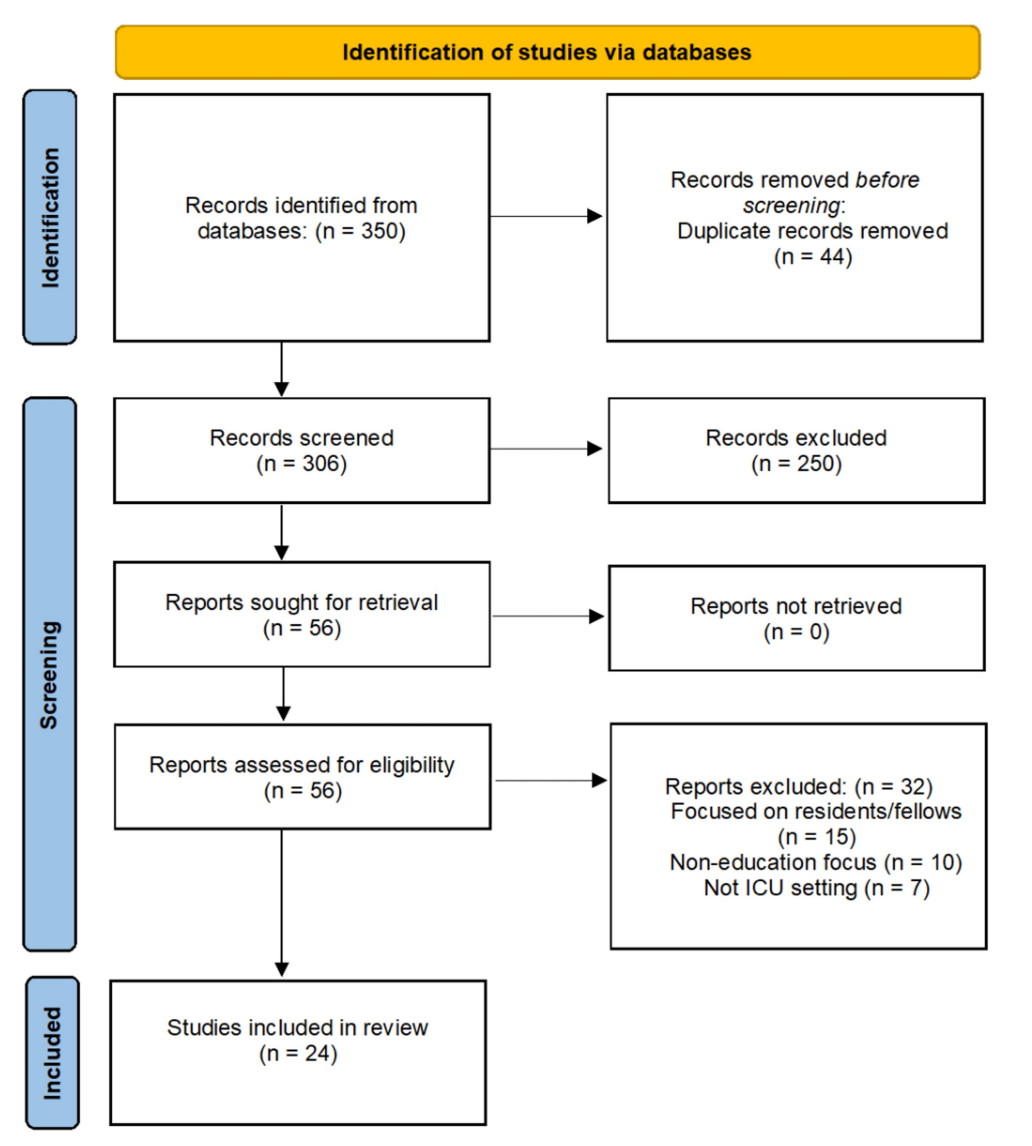
PRISMA flow chart of study selection process PRISMA - Preferred Reporting Items for Systematic Reviews and Meta-Analyses

All retrieved records were imported into a reference manager. After removing duplicates, titles and abstracts were screened independently by two reviewers. Full texts of potentially eligible studies were reviewed in full. Discrepancies were resolved by consensus. A total of 350 articles were initially identified based on the search parameters. After removing duplicates, there were 306 total articles left that were to be further reviewed. Based on titles and abstracts, another 250 studies were excluded, leaving a total of 56 articles to be analyzed. After full-text review, only 24 articles were selected for this study. The selection process is presented in the flow chart in Figure 1.

Data Extraction

Data were extracted from each included study using a standardized form designed to capture key characteristics relevant to ICU-based medical education for medical students. A standardized data extraction form was used to chart information including study design, research aims, sample size, ICU type, teaching intervention details, learner level, outcomes measured, and key findings. This process was conducted independently by two reviewers to ensure accuracy and consistency. Discrepancies between reviewers were resolved through discussion and consensus. Findings were grouped and summarized using a narrative synthesis approach, with thematic categorization based on intervention type and reported educational outcomes.

Results

A total of 24 studies were selected for this scoping review and summarized in Table 1. Study designs included seven cross-sectional surveys, five intervention-based studies (e.g., pre-post or quasi-experimental), four curriculum development or evaluation papers, three literature or systematic reviews, two qualitative studies, and three descriptive or feasibility studies. Educational interventions varied, with simulation-based training featured in 10 studies, flipped classroom models in four, tele-ICU or virtual formats in three, peer-assisted learning in three, and traditional didactics or bedside teaching approaches in four. Most studies involved third or fourth-year medical students participating in clinical electives, required clerkships, or simulation-based modules. A smaller number of studies included emergency medicine or anesthesiology residents.

**Table 1:** Selected study articles (n=24)

Article title	Author(s)	Year	Study method	Aim	Major findings
Reimagining Undergraduate Critical Care Medical Education: A Path for the Next Decade	Gergen et al. [1]	2024	Perspective/review	Propose future ICU curricular strategies	Advocates for widespread, multimodal ICU integration
Integrated Critical Care Curriculum for the Third-Year Internal Medicine Clerkship	Gergen et al. [2]	2020	Curriculum development	Integrate ICU learning into clerkship	Improved exam scores and high student satisfaction
A Blueprint for Improving Undergraduate Education in Intensive Care Medicine	O'Connor & Martin‑Loeches [6]	2016	Commentary/strategy	Propose guidelines to enhance ICU teaching	Recommended structured curriculum and faculty training
Development of an Undergraduate Medical Education Critical Care Content Outline Utilizing the Delphi Method	Smith et al. [7]	2020	Delphi consensus	Define essential ICU competencies	Resulted in a standardized curriculum framework for student ICU content
Intensive Care Medicine on Medical Undergraduation: Student's Perspective	Almeida et al. [8]	2007	Survey	Assess ICU exposure and perception in Brazil	Most students had little to no ICU exposure and perceived this as a gap
A Qualitative Study of Undergraduate Clerkships in the Intensive Care Unit: It's a Brand New World	O'Connor et al. [9]	2017	Qualitative interviews	Explore clerkship student experiences	Students experienced initial anxiety but gained agency
Feasibility Study of a Fully Synchronous Virtual Critical Care Elective Focused on Learner Engagement	Hwang et al. [10]	2022	Feasibility study	Test virtual ICU elective model	High satisfaction and boosted learner confidence
Assessing the Impact of Simulation-Based Learning on Student Satisfaction and Self-Confidence in Critical Care Medicine	Ageel et al. [11]	2024	Pre‑post intervention	To assess how simulation affects ICU preparedness	Increased confidence, satisfaction, and clinical skills post-intervention
Simulation-Based Team Training in Time-Critical Clinical Presentations in Emergency Medicine and Critical Care: A Review of the Literature	Weile et al. [12]	2021	Literature review	Review team simulation in ICU and ER settings	Simulation enhances decision, teamwork, and leadership
The Impact of Simulation-Based Training in Medical Education: A Review	Elendu et al. [13]	2024	Narrative review	Examine simulation's role in med ed	Simulation shown to increase skills and confidence across disciplines
Peer-Assisted Learning in Critical Care: A Simulation-Based Approach for Postgraduate Medical Training	Chiu et al. [14]	2025	Systematic review	Assess impact of peer‑led ICU simulation	Enhanced teamwork, leadership, and communication skills
Enhancing Critical Care Training through Simulation-Based Education: A Comparative Study	Ma et al. [15]	2024	Comparative study	Compare simulation vs traditional methods	Simulation significantly increased preparedness and teamwork
Evaluating Student Satisfaction and Self-Confidence in Simulation-Based Anesthesiology Training among Final-Year Medical Students	Shbeer [16]	2024	Pre‑post intervention	Evaluate anesthesia simulation's effect	Increased satisfaction and self-confidence in critical skills
Weekly Flipped Classroom Modules in Intensive Care Medical Training: Feasibility and Acceptance	Scholte & Strehler [17]	2025	Pilot program	Test flipped classroom in ICU rotation	High feasibility, engagement, and knowledge retention
Physiology Education for Intensive Care Medicine Residents: A 15-Minute Interactive Peer-Led Flipped Classroom Session	Zante et al. [18]	2020	Peer-led flipped classroom	Teach ICU physiology through flipped peer teaching	Greater understanding and enthusiasm reported
From Physiology to Clinical Practice: Integrating Peer-Teaching and Team-Based Learning in Intensive Care Medicine	Zante et al. [19]	2021	Program evaluation	Combine peer-led and TBL in ICU	Improved team engagement and clinical reasoning
Effectiveness of an ICU-Focused Flipped Classroom Model on Knowledge Retention and Clinical Preparedness	Zhang et al. [20]	2023	Quasi‑experimental	Test flipped classroom for ICU learning	Enhanced knowledge retention and clinical preparedness
Developing the eMedical Student (eMS)-A Pilot Project Integrating Medical Students into the Tele-ICU during the COVID-19 Pandemic and beyond	Ho et al. [21]	2021	Descriptive study	Describe tele-ICU elective experiences	Students reported improved comfort and tele‑ICU skillsets
Knowledge and Attitudes Regarding Do-Not-Resuscitate Decisions Among Clinical-Year Medical Students and Interns in the Western Region of Saudi Arabia	Binjabi et al. [22]	2024	Survey	Examine student attitudes on end-of-life ICU decisions	Many students lacked experience and confidence in do not resuscitative (DNR) scenarios
Effectiveness of the Introduction to Critical Care in Emergency Medicine Curriculum's Implementation Among Trainees Interested in Intensive Care	Carvey et al. [23]	2023	Program evaluation	Evaluate hybrid critical care training course	Students showed improved knowledge and decision-making
Comparison of Knowledge and Confidence Between Medical Students as Leaders and Followers in Simulated Resuscitation	Vattanavanit et al. [24]	2020	Prospective simulation-based study	Compare knowledge and confidence between student leaders and followers in ICU simulations	Both roles showed similar gains in knowledge and confidence after simulation training
Knowledge and Confidence of Final-Year Medical Students Regarding Critical Care Core-Concepts, a Comparison between Problem-Based Learning and a Traditional Curriculum	Al Ansari et al. [25]	2021	Cross-sectional survey	Compare knowledge/confidence across curricular formats	Problem Based Learning (PBL) students scored higher and felt more confident in ICU concepts
Knowledge and Competence Towards Critical Care Concepts Among Final Year Medical Students and Interns: A Cross-Sectional Study	Dairi et al. [26]	2022	Cross-sectional	Assess student critical care competence	Low knowledge levels and self-perceived ability
The Impact of Current Experience, Level of Training, and Post-call Status on Student and Resident Examination Results During a Surgical ICU Rotation	Seely et al. [27]	2001	Observational	Assess ICU rotation effect on learning	Rotation improved knowledge gains vs wards initially

**Figure 2:**
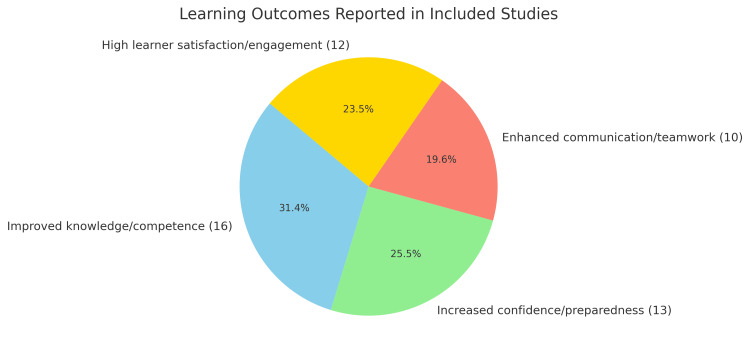
Learning outcomes reported in included studies Among the 24 studies reviewed, 12 studies showed medical students had higher learning satisfaction/engagement, 10 studies showed medical students had enhanced communication/teamwork skills, 13 studies showed medical students had increased confidence/preparedness, and 16 studies showed medical students have improved knowledge/competence all after completed an ICU rotation.

**Figure 3:**
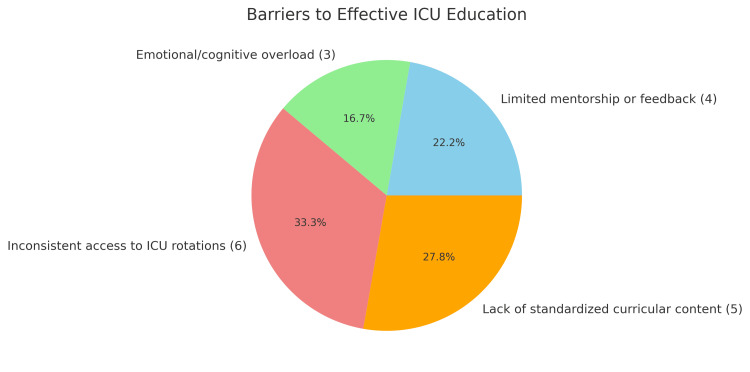
Barriers to effective ICU education Among the 24 studies reviewed, barriers to effective ICU education included: inconsistent access to ICU rotations (six studies), a lack of standardized curricular content (five studies), emotional or cognitive overload among students (three studies), and limited mentorship or feedback (four studies)

Regarding outcomes, 16 studies reported improvements in student knowledge or clinical competence, 13 described increased learner confidence or preparedness for ICU settings, and 10 noted enhanced communication, teamwork, or leadership skills. Learner satisfaction and engagement were positively reported in 12 studies, particularly where interactive or structured formats were used (Figure 2). Key barriers to effective ICU education included inconsistent access to ICU rotations (noted in six studies), a lack of standardized curricular content (five studies), emotional or cognitive overload among students (three studies), and limited mentorship or feedback (four studies) (Figure 3). These findings highlight a growing commitment to improving critical care education for medical students and suggest the need for broader implementation of structured, competency-based ICU curricula.

Importance of ICU Training in Medical Education

Multiple studies underscore the critical role of ICU exposure in shaping competent new physicians. Critical care units offer unique learning opportunities, such as exposure to severe pathology, rapid clinical decision-making, and interdisciplinary team dynamics, that are not fully replicated in other rotations [6]. ICU training bridges fundamental physiology to clinical application, reinforcing concepts like hemodynamics, ventilation, and multi-organ support [3]. Consequently, there is growing consensus that all medical graduates should attain basic competencies in recognizing and managing acutely ill patients prior to residency [6]. However, actual practice lags behind this idea. Surveys indicate that fewer than half of medical schools (in the United States, for example) mandate an ICU rotation for students [2]. A 2020 national Delphi consensus in the US confirmed the lack of a standardized critical care curriculum in undergraduate medical education, and this study produced a content outline to address this gap, highlighting that basic recognition of critical care medicine and initial stabilization skills are the priority for new graduates [7].

Importantly, early critical care exposure appears to confer lasting benefits. A recent perspective noted that early ICU training can lessen students' anxiety in acute situations while boosting their confidence and medical knowledge [3]. Students themselves recognize this value. In a Brazilian survey, 97% of medical students agreed that ICU topics should be more thoroughly taught in their curriculum [8]. In this study, it showed that over half of the medical students participating had never set foot in an ICU during training, yet interest in critical care was high (54% expressed high/very high interest). The lack of ICU experience clearly affected self-efficacy with only 42% feeling able to assess a critically ill patient, whereas those who had completed an ICU elective were significantly more comfortable (p<0.001) [8]. These findings collectively highlight that ICU training is both desired by learners and necessary for competence, and its absence leaves a notable educational gap. The importance is further reinforced by professional guidelines. The Association of American Medical Colleges included acute care of unstable patients as an Entrustable Professional Activity (EPA 10) expected of all graduates, a competency that essentially mandates some critical care training [3]. Overall, the literature strongly supports making ICU exposure a standard part of graduate medical education to ensure new physicians can "assume appropriate responsibility after graduation" in caring for critically ill patients [6].

Overall, findings from the 24 study articles used in this scoping review are presented across three key domains: teaching methods used in ICU education, learning outcomes of ICU education, and gaps identified in current literature and ICU medical training.

Teaching Methods Used in the ICU

Academic clinicians have employed a variety of different teaching methods to integrate critical care knowledge and skills to medical students. Given the ICU's fast-paced, hands-on environment, it was a consistent theme in the articles that the traditional lecture-based didactics alone were not sufficient. To maximize learning, successful ICU curricula incorporated multiple modalities, including bedside teaching, simulation, case-based discussions, and more. Key teaching strategies reported in the literature included:

Bedside and rounds-based teaching: Bedside teaching during ICU rounds remains cornerstone to medical education. Students actively participate in daily rounds, learning to present complex cases and formulate management plans under direct supervision. In surveys of residents reflecting on medical school, many felt that more rounds-based teaching in ICU would have better prepared them for residency training [6]. Bedside teaching in the ICU not only reinforces clinical reasoning but also allows observation of procedures and communication with families at bedside. It's been shown that medical students felt that ICU clerkship differed from ward rotations as the students often initially feel "unfamiliar with the environment and complex illness", but they navigate through phases of learning and gain their own agency after participating more in guided rounds. Key factors aiding learning for medical students were supportive ICU staff and seeing real-time clinical decision-making [9]. However, bedside teaching can be variable. Heavy service responsibilities and demands can limit personalized teaching time in the ICU. Programs have found a solution to this by scheduling dedicated teaching rounds or using structured daily goal sheets for students to fill out on each patient, which focuses their attention during rounds and improves engagement [10]. Altogether, while bedside teaching in the ICU presents challenges, structured support, like dedicated teaching rounds and goal-oriented tools, can enhance student involvement, helping them transition from observers to active participants in patient care.

Simulation-based teaching: Simulation has emerged as a powerful tool in critical care education. High-fidelity simulations allow students to practice managing emergencies (like cardiac arrest, shock, or respiratory failure) in a realistic yet controlled setting. Numerous studies demonstrate that simulation-based training significantly enhances clinical skills, confidence, and preparedness [11,12]. In critical care contexts, simulation gives learners exposure to acute scenarios that may be rare or too high-stakes to encounter as a student. For example, Ageel (2024) implemented simulation-based learning (SBL) sessions on ICU scenarios for final-year students and found marked improvements in self-reported outcomes. After the SBL intervention, students' satisfaction and self-confidence scores in critical care learning were significantly higher (mean ~4.2 on five-point scales) compared to before [11]. A strong positive correlation was noted between satisfaction and confidence, underscoring that well-designed simulations both engage students and build their self-efficacy [11]. Simulation allows repetitive practice and immediate feedback, which solidifies knowledge and skills. Skills learned via high-fidelity simulation have been shown to better retain over time than those taught by lecture alone [13]. Importantly, simulations also improve non-technical skills such as teamwork and communication in a crisis [12]. One innovative approach in Taiwan integrated peer-assisted learning with simulation. Junior trainees alternated roles as team leaders and observers in simulated ICU emergencies, then provided peer feedback. This method significantly boosted participants' clinical confidence, teamwork, and leadership abilities in critical care settings. The collaborative nature of peer simulation debriefings created a safe space for reflection and was highly rated by learners [14,15]. Even outside the ICU, simulation is being used to teach related acute care skills. For instance, a study of simulation-based anesthesiology training for final-year medical students reported high satisfaction and confidence gains similar to those seen in critical care simulations [11,16]. Taken together, simulation is a core modality in modern ICU education, offering hands-on practice in managing critical events without risk to patients.

Flipped classrooms and case-based learning: Given the complexity of ICU medicine and time constraints on-site, some programs have adopted a flipped classroom model. In a flipped ICU curriculum, students complete online preparatory modules or readings on critical care topics in advance, often with quizzes or "control questions", and then spend classroom or ICU time on active learning (case discussions, problem-solving, etc) facilitated by the attending faculty [17]. Scholte and Strehler (2025) piloted weekly flipped classroom modules during an ICU rotation and found it highly feasible and well-received. All medical students accessed the online content (with 78% average participation), and those learning through the flipped model showed significantly greater motivation and perceived benefit than prior cohorts [17]. Learning motivation scores improved from 19.0 pre-flip to 23.8 post-flip (p<0.001) [17]. Trainees especially appreciated the flexibility and the blend of online materials with in-person discussion. Similarly, purely case-based, small-group sessions have been integrated into clerkships.

At one US medical center, instructors created a seven-session ICU case conference series within the internal medicine clerkship, covering shock, respiratory failure, sepsis, and more [2]. These sessions were co-led by critical care faculty and residents, with interactive discussion of clinical cases. This case-based curriculum led to increased objective knowledge gains, post-test score improvements from 50% to 71% (p<0.0001), and students overwhelmingly felt it enhanced their overall clerkship experience. Notably, 95% of students in that program agreed their comfort in managing critically ill patients had increased [2]. Zante et al. (2020) demonstrated the effectiveness of a peer-led, 15-minute flipped classroom session focused on core physiology concepts in intensive care, showing that even brief, interactive modules can significantly improve learner engagement and knowledge retention [18]. In a follow-up study, Zante et al. integrated peer teaching with team-based learning in an ICU education setting, finding that this combined approach enhanced clinical reasoning, collaboration, and learner satisfaction [19]. One final benefit from the flipped classroom approach is the effectiveness of learning. Zhang et al. (2023) evaluated an ICU-focused flipped classroom model and found it significantly improved both short-term knowledge retention and students' self-reported preparedness for clinical duties in critical care environments [20]. This study also emphasized the importance of pre-session materials and interactive case discussions in reinforcing core ICU concepts and fostering clinical confidence. Overall, the flipped classroom approach was seen as a flexible, learner-centered strategy that helped bridge the gap between theoretical knowledge and ICU bedside practice [20]. In the end, all these findings illustrate that even if a full ICU rotation is short or unavailable, targeted case-based teaching can effectively introduce key ICU principles, especially for flipped-based classrooms or case-based teaching.

Digital and remote learning: The COVID-19 pandemic spurred innovations in virtual ICU education. When in-person rotations were suspended, educators implemented fully online ICU electives and telemedicine experiences. One feasibility study from 2020 described a two-week virtual critical care elective where students joined ICU rounds via video conferencing (using a head-mounted camera worn by the ICU attending) [10]. Students remotely participated in multidisciplinary rounds and completed daily electronic ICU goal sheets for each patient. Despite being entirely virtual, engagement was high, and the medical students logged hundreds of hours of active participation and found the structured goal sheets helpful for following patient progress. Feedback was overall positive with 88.9% of students agreeing that the virtual elective improved their comfort in caring for critically ill patients [10]. Another program integrated students into a tele-ICU service monitoring on COVID-19 patients. In this three-week pilot, students remotely contributed to patient management (with attending oversight) and made ~70 clinical recommendations (e.g., ventilator and medication adjustments) throughout the service time [21]. Students reported increased confidence in managing critically ill patients and in using telehealth technology, all while avoiding infection risk. The authors concluded that tele-ICU rotations are a safe, innovative way to continue critical care education and could be adapted even beyond the pandemic [21]. Additionally, online open-access critical care education resources (blogs, video series, etc.) have proliferated (often termed FOAMed, Free Open Access Medical education). These digital modalities, while relatively new, appear to supplement traditional learning and help overcome scheduling and geographic barriers in ICU training. Overall, the rapid shift to digital formats during the pandemic revealed that virtual ICU education, whether through tele-ICU experiences or open-access online resources, can effectively complement traditional training, expand access, and enhance student engagement in critical care learning.

In summary, ICU educators are adopting a multimodal approach to teaching and educating medical students. Effective ICU teaching uses a mix of various teaching modalities and even increasingly utilizes online or virtual tools [23]. This blended strategy caters to diverse learning styles and maximizes exposure to critical care situations. The literature indicates that medical students value these interactive, hands-on, and diverse teaching methods, and they lead to improved competence compared to lecture-only didactics.

Learning Outcomes of ICU Education for Medical Students

Studies assessing ICU-focused education reported a variety of beneficial outcomes for learners. These outcomes can be grouped into knowledge and skills acquisition, self-confidence and preparedness, attitudinal changes and learner satisfaction, and career impact and specialty choice.

Knowledge and Skills Acquisition

Almost every study reviewed noted a measurable improvement in medical students' knowledge or skills related to critical care after an ICU rotation. For instance, the integrated ICU curriculum by Gergen et al. yielded a 21% absolute increase in knowledge test scores after the ICU rotation was completed (from 50% to 71%) [2]. Simulation training likewise has been shown to improve performance on skills checklists. In Saudi Arabia, final-year medical students who underwent an ICU simulation course demonstrated higher competency in assessing and stabilizing patients compared to those without ICU simulation exposure [11]. A broad 2024 review on simulation in medical education concluded that simulation-based learning leads to enhanced skill acquisition and error reduction, ultimately translating into better patient care practices [13]. Some evidence suggests these gains are retained over time with reinforcement, though true long-term retention remains a gap in research. Nevertheless, immediate post-training assessments consistently favored those who had ICU-focused teaching over controls. Vattanavanit et al. (2020) implemented high-fidelity simulation scenarios involving shock resuscitation to evaluate leadership and teamwork roles among medical students in a controlled ICU-like setting. Structured debriefing sessions following each simulation were highlighted as essential for reinforcing clinical reasoning and team-based communication [24]. Even brief interventions mattered. One study reported that a short, one-day critical care course for medical students produced significant improvement in their ICU knowledge test scores, highlighting that even limited ICU training can yield tangible knowledge gains if well-designed [28]. These findings underscore that even short, focused ICU educational experiences, especially those involving simulation and structured feedback, can lead to meaningful and measurable improvements in students' critical care knowledge and clinical skills.

Self-Confidence and Preparedness

A major outcome reported from the studies is the increased confidence in managing critically ill patients after medical students participated in an ICU rotation. Because critical care situations can be intimidating, building student confidence is important. Many studies rely on self-reported confidence scales. The trend is clear. ICU exposure improves confidence. In the flipped ICU module study, learners' self-rated preparation and motivation for ICU tasks rose significantly after the intervention [17]. Ageel's 2024 study found that after simulation, students' confidence in critical care learning was not only higher on average, but strongly correlated with their satisfaction in training [11]. Students commonly cite ICU rotations as transformative for their self-efficacy: facing high-acuity cases under guidance makes them feel more ready to handle emergencies. Even virtual ICU electives during COVID improved medical students' comfort with critical care management (nearly 89% felt more comfortable) [10]. Additionally, studies have demonstrated significant improvements in both knowledge and self-confidence among all medical students participating in the ICU-simulation exercises, regardless of whether they served as team leaders or followers [24]. Importantly, those who lack ICU education report low confidence. The survey by Al Ansari et al. (2021) showed that the vast majority of students without substantial ICU exposure did not feel confident recognizing shock or respiratory failure [25]. This was echoed in the Saudi study, where over 75% of final-year medical students did not feel competent performing basic ICU airway skills [25]. Thus, ICU training can dramatically shift students from a state of low confidence to a state of greater readiness. It's notable that confidence does not equate to competence, but in these studies, objective improvements accompanied the subjective gains. For example, in the MedEdPORTAL ICU curriculum, students' confidence in participating in care correlated with their improved test scores [2]. Confidence is also linked to reduced anxiety; early ICU training has been noted to lessen students' anxiety about acute care situations, which likely makes them more effective under pressure [3]. Together, these findings suggest that ICU education plays a critical role in bridging the gap between theoretical knowledge and clinical readiness, helping students feel more capable, less anxious, and better prepared to care for critically ill patients.

Attitudinal Changes and Learner Satisfaction

Students consistently reported high satisfaction with ICU learning experiences, especially when active learning methods were used. Simulation-based sessions have garnered excellent feedback, for example. In Ageel's study, satisfaction scores were very high (mean ~3.7-4.2/5) and strongly tied to increased self-confidence [11]. Students appreciated the "safe environment" to practice and the realism of the various scenarios. Similarly, in flipped classrooms, trainees valued the flexibility and quality of materials, which led to an increase in their motivation to learn ICU topics and critical care management [17]. Qualitative feedback often highlights that ICU rotations teach not just clinical management, but also focus on teamwork, communication, and systems-thinking (e.g. triage, resource use, decision making, etc.) [26]. Students frequently mentioned that ICU experiences, though challenging, are among the most educational rotations during medical school [9]. On the other hand, medical schools where ICU teaching was minimal, students expressed a desire for more. In one Brazilian survey, virtually all students felt critical care medicine should be emphasized more during their training [8]. Such attitudes have driven the creation of student-led critical care interest groups and electives in some medical schools. These findings collectively suggest that meaningful exposure to ICU education not only builds clinical skills and confidence, but also sparks long-term interest in critical care among students.

Career Impact and Specialty Choice

An interesting outcome is whether ICU exposure influences students' career choices or residency preparedness. Some evidence suggests it can increase interest in critical care or related fields. Early exposure has been correlated with higher likelihood of pursuing additional critical care experiences during medical training. For instance, after an integrated ICU curriculum, more medical students chose to take a fourth-year ICU elective or indicated interest in an ICU fellowship down the line [3]. However, data on ultimate career choice is mixed. Al Ansari et al. found only 6.5% of surveyed Saudi medical students planned to pursue critical care as a career, indicating that despite increased knowledge, other factors influence specialty decision [25]. Nevertheless, ICU training undoubtedly makes students more comfortable entering any residency. Program directors have noted that intern physicians with prior ICU experience transition more smoothly to caring for very sick patients [6]. Additionally, ICU rotations may instill attitudes of multidisciplinary collaboration and awareness of end-of-life care that benefit future practice in any medical field [26]. A few studies also looked at performance outcomes. Seely et al. (2001), albeit older data, observed that medical students' knowledge scores improved in a dose-dependent fashion with ICU rotation length (plateauing around 6-7 weeks of exposure) and that shorter rotations might capture only part of the potential learning curve [27]. While extended ICU rotations for students are rare, this finding implies that more longitudinal or repeated ICU experiences could yield even greater competence. These findings suggest that while ICU exposure may not directly drive most students toward a critical care career, it plays a valuable role in shaping professional development, improving residency readiness, and reinforcing core skills that are applicable across medical specialties.

In summary, the outcomes of ICU-based education are overwhelmingly positive for learners. In the ICU, medical students acquire critical medical knowledge, improve procedural and cognitive skills, and gain confidence in managing acute illnesses. They also develop a better appreciation for the complexity of care and importance of communication and teamwork. High satisfaction with ICU learning experiences is nearly universal when programs are organized and well-structured. The only caution is that many studies measure immediate or short-term outcomes (end-of-rotation tests, surveys, etc) and not long-term retention or patient-level outcomes. Still, the consistency of improved exam scores and self-confidence across diverse settings (US, Europe, Middle East) indicates that ICU education adds significant value to medical training.

Gaps in the Current Literature and Medical ICU Training

Despite progress in integrating critical care into medical education, the literature reveals several important gaps and underrepresented areas in ICU education and teaching during medical school.

Geographic Disparities

There is uneven research coverage and likely variability in ICU training practices across regions. A large portion of published studies in the last decade come from high-income countries (US, Canada, Western Europe) and from select Middle Eastern countries (notably Saudi Arabia) [25,26]. These studies often highlight local ICU curriculum changes or needs. In contrast, there is a relative paucity of data from low and middle-income countries in Africa, South Asia, and Latin America. It is likely that ICU exposure is even more limited in many of these regions' medical schools, but documentation is scant. An older Brazilian study (2007) illustrated a substantial gap in that setting, where most students had no ICU rotation but desired one in their medical school curriculum [8]. More recent data from other Latin American or African nations are not well-represented in the literature. This points to the need for wider international collaboration and studies to understand how critical care is taught globally. Additionally, within countries, there may be disparities, for example, in the US, some medical schools have robust ICU clerkships while others offer only electives or none at all [2]. Irish educators reported "regional variability" even among their few medical schools, with some having mandatory ICU weeks and others none [6]. Such variability likely exists in other countries as well, suggesting that many students worldwide still graduate without adequate critical care exposure. ICU education shows significant geographic and institutional variability, with limited data from many low- and middle-income regions, underscoring the need for broader, global research efforts to better understand and address gaps in critical care training worldwide.

Lack of Standardization and Curriculum Development

As noted, there was no standardized consensus on curriculum for critical care medicine at the medical school level. The Delphi study in 2020 was a first step to try and outline core contents for the ICU [7]. Prior to that, ICU topics were often taught ad hoc by individual instructors, leading to heterogeneity in what students learn. One letter observed that even in ICUs that take students, one-third had no formal curriculum or objectives, and nearly all had no formal assessment or evaluation for students [6]. This lack of structure can result in variable educational quality. Additionally, many ICU rotations are very short (often one to two weeks), which may limit the depth of learning unless a focused curriculum is in place [6]. The literature calls for better curriculum design with clear learning objectives, combining bedside and didactic methods, and incorporating the evaluation of student performance [6]. Some recent innovations, like the consensus topics list or the use of structured assessment tools (daily goal sheets, case presentations, simulation checklists), aim to bring more rigor to ICU education [7]. However, widespread adoption of a standard curriculum is still lacking. This gap suggests an opportunity for academic and critical care societies to develop and disseminate curriculum guidelines and perhaps certificate courses. For example, the Society of Critical Care Medicine's Fundamental Critical Care Support (FCCS) course has been adapted for students in some places [1]. Standardization would also facilitate multi-center studies on outcomes, which are currently difficult due to inconsistent curricula. The absence of a standardized ICU curriculum at the medical school level leads to inconsistent training quality, highlighting the need for clear learning objectives, structured assessments, and wider adoption of consensus guidelines to improve and unify critical care education.

Outcome-Based Research Deficiency

Many studies measure learners' self-reported confidence or knowledge via tests. While test scores are important, there is a gap in outcome-based studies that connect ICU training to downstream effects. For instance, do medical students with ICU rotations perform better in internship (fewer medical errors, better patient outcomes) compared to those without? Such hard outcomes are not well studied. One reason is the challenge of attribution and long-term tracking. However, a few clues exist. A study in Brazil noted that students with ICU experience felt more secure in patient care decisions, which likely translates to better performance as new doctors, though direct patient impact was not measured [8]. Another gap is long-term knowledge retention. It is unknown how much of the critical care knowledge gained in medical school is retained into residency a year or more later. General literature on skill decay suggests that without reinforcement, clinical skills can deteriorate within months [13]. If ICU concepts are not revisited, students might lose some proficiency. No identified studies specifically re-tested students many months after an ICU course to assess retention. Addressing this gap might require integrating refreshers or bridging activities, for example, a revisit of critical care topics during internship orientation. Additionally, while improved confidence is frequently reported, it would be valuable to confirm that this confidence is matched by competence in real clinical encounters as trainees. Overly confident but under-trained resident physicians could be risky. So far, the alignment of subjective and objective outcomes in studies like Gergen 2020 is reassuring, but more robust competency assessments (e.g., ICU OSCEs) could further strengthen outcome evaluation [2]. Overall, while existing studies show promising gains in knowledge and confidence, future research should focus on linking ICU education to real-world performance, long-term retention, and objective measures of competence to better understand its true impact on clinical readiness.

Underrepresented Topics

The current literature has heavily focused on acute management skills (airway, shock, sepsis, etc) and technical and procedural competencies. Far less is published about teaching soft skills and interdisciplinary aspects in the ICU. Communication and end-of-life care training is one such underrepresented area. ICU care often involves goals-of-care discussions, breaking bad news, and palliative care integration. A few studies note that medical students rarely get to actively participate in these conversations as learners. Consequently, new doctors feel less prepared for end-of-life communication with patients and families [22]. One recent study in Saudi Arabia by Binjabi et al. (2024) assessed knowledge and attitudes toward do-not-resuscitate (DNR) orders among final-year medical students. It found notable gaps where many students had only theoretical knowledge of DNR and had never observed an ICU family meeting [22]. The authors highlighted the need for better education in end-of-life decision-making for medical students. Another under-taught topic in the ICU is ethics and resource allocation. Hardly any medical school curricula formally cover this. Psychosocial aspects like handling stress, burnout, and communicating with ICU families are also seldom addressed in medical student rotations. Furthermore, while teamwork is inherent in ICU care, formal interprofessional education (with nurses, respiratory therapists, etc.) for medical students in the ICU is not well documented. Simulation can incorporate teamwork elements, but also real-world interprofessional ICU learning for students is an area for growth. In short, while technical skills dominate ICU curricula, there's a clear need to expand education around communication, ethics, end-of-life care, and interprofessional teamwork to better reflect the full scope of ICU practice and prepare students for the complex realities of critical care.

Faculty and Resource Constraints

Some gaps in the implementation of ICU education are highlighted by restraints of faculty and hospital resources. Commonly reported barriers to teaching students in the ICU include limited faculty time, the intensity of patient care and responsibilities, and concerns that learners might slow down workflow. In a survey, ICU educators cited insufficient staffing and lack of protected teaching time as major impediments to student teaching [6]. These systemic issues mean that even if a curriculum exists on paper, delivering it consistently is challenging. Innovative solutions (like involving senior residents in teaching roles or using online modules to cover knowledge so that faculty can focus on bedside teaching) are being trialed, but more research on how to best overcome these constraints would be beneficial. The literature also lacks studies evaluating faculty development for ICU teaching. Training intensivists or fellows on how to teach effectively in an ICU environment could be valuable, yet few papers touch on it. Overall, these challenges highlight that despite well-designed curricula, limited faculty availability, and competing clinical demands often hinder consistent ICU teaching, pointing to a need for creative approaches and better support for educators to ensure effective student learning.

In summary, while there have been many advances in ICU medical education, key gaps continue to remain. Broader international data is required to ensure every medical student, regardless of location, gets adequate critical care exposure. There is a need to standardize both the teaching content delivered and the outcomes measured, ensuring that assessments capture not only immediate learning but also long-term outcomes and competency development. Additionally, expanding the scope of ICU education to include communication, ethics, and telemedicine will be important. Addressing these gaps will require targeted efforts and collaboration between academic medical centers and critical care societies to develop guidelines, share resources, and advocate for ICU training as a core element of medical education.

Discussion

This scoping review confirms that integrating critical care medicine into medical school is both highly important and largely beneficial, but it also reveals inconsistencies in implementation across the globe. The intensive care unit offers a "rich, social learning environment" distinct from other rotations, one that teaches acute clinical acumen, procedural skills, and interdisciplinary teamwork under pressure [9]. Students who experience ICU training consistently demonstrate better knowledge of critical care, improved clinical skills, and greater confidence in handling severely ill patients. These outcomes align with the fundamental goal of medical education, which is to produce physicians who are ready to assume responsibility for patient care on day one of residency [6].

One striking finding is the mismatch between the recognized value of ICU training and its current prevalence in curricula. Multiple sources emphasize that all future doctors should have some critical care competency, yet it was found that many medical schools still do not offer formal ICU rotations or robust ICU content [1,6]. In settings where ICU exposure is optional or absent, graduating students report significant knowledge and confidence gaps [25,26]. This suggests that medical schools that have not yet incorporated ICU training are likely leaving their students less prepared for critical patient care, potentially at the expense of patient safety and the interns' own anxiety when they face their first crashing patient. Bridging this gap may require educational policy changes. Just as most schools require rotations in medicine, surgery, pediatrics, etc., there is a growing argument to require at least a short critical care experience. Where logistical constraints (e.g., limited ICU capacity for learners) exist, alternatives such as simulation-based courses or "ICU bootcamps" can serve as substitutes [28]. Even short courses and simulations effectively impart critical care fundamentals to medical students. Schools in resource-limited settings might implement a simulation-plus-discussion curriculum to achieve learning outcomes even if an in-person ICU rotation is not feasible for all students.

This review also highlights effective educational strategies that can be models for curriculum development. Traditional passive learning in ICU (or mere observation) is inadequate. Instead, active learning approaches yield the best results, such as simulation drills for emergencies, flipped classrooms for theory, hands-on involvement in patient care, and case-based reflection sessions. The combination of methods addresses the multi-dimensional learning needed, which includes cognitive (knowledge), psychomotor (skills), and affective (confidence, teamwork). For instance, the use of peer-assisted simulation not only taught clinical management but also improved communication and leadership among trainees [14]. Programs aiming to introduce ICU concepts might consider adopting a mixed modality structure. An example such as a preparatory e-learning module (to cover basics like ventilator modes, sepsis protocols), followed by an intensive week of simulation and bedside rounding in an ICU, capped with reflective debriefs. Such a structure could maximize learning in a short time frame, an approach that seemed to work in the studies reviewed [2].

Another key discussion point is the importance of evaluation and feedback in ICU education. It was observed that many ICU rotations historically lacked formal assessment of students [6]. However, introducing evaluations (even if formative) can greatly enhance the learning process. The daily goal sheet used in a virtual ICU elective, for example, functioned as both a learning tool and an engagement metric [10]. Students knew they would articulate plans for each patient, which kept them mentally active during rounds. Similarly, post-rotation knowledge tests or simulation-based competency assessments gave students concrete feedback on their performance and gave educators data to refine curricula. As critical care curricula become more defined (with consensus topics and objectives), aligning assessments to these objectives will be important [7]. It not only reinforces learning but also signals to students that critical care knowledge is a core expectation, not an ancillary elective information.

Additionally, this review identifies several areas in need of further research and development. One is the longitudinal outcomes. It would be useful to follow cohorts of students with and without ICU training into residency to see differences in performance or patient outcomes. Also, the optimal timing and duration of ICU exposure merits discussion. Many of the described ICU rotations occur in the final year of medical school (acting internships, etc.), which makes sense for readiness just before residency. But some argue that introducing critical care earlier (e.g., brief exposures in second or third year) could reinforce basic science learning and spark interest early [3]. The concept of a spiral curriculum could apply by revisiting critical care themes at increasing levels of complexity over the course of medical school. The consensus content list (19 topics) could potentially be distributed throughout existing courses (e.g., shock in cardiovascular module, acute respiratory distress syndrome in pulmonary module, etc.), with a capstone ICU rotation tying them together [7]. This integrated approach might ensure better retention and understanding. However, no studies have directly compared an integrated curriculum versus a single rotation. This could be fertile ground for education research. Overall, these gaps point to the need for long-term studies tracking the impact of ICU education on residency performance, as well as exploration of earlier and more integrated critical care teaching models to enhance knowledge retention and engagement throughout medical school.

Geographically, it is clear that context-specific solutions are needed. In resource-rich environments, incorporating simulation centers and dedicated ICU electives is realistic, whereas in resource-limited settings, creative approaches like leveraging experienced clinicians to deliver Fundamental Critical Care Support courses or using low-cost simulation (even role-play and basic mannequins) might be more feasible. The positive results from short courses (one to two days) in knowledge gains are encouraging. They suggest that even a brief intervention can be impactful if carefully planned [28]. International collaborations, such as global critical care education exchanges or sharing of open-access materials, could help schools with fewer resources implement some form of ICU training. With the expansion of tele-education (as seen during COVID), one could envision regional virtual ICU teaching sessions where an intensivist could teach students from multiple institutions remotely. The findings from virtual electives indicate that student engagement and learning can be maintained in an online format if done synchronously and interactively [10].

Finally, this review draws attention to underemphasized competencies such as interdisciplinary collaboration and communication in ICU settings. As medical education shifts toward competency-based frameworks, we should ask: Are we training students not just to run a code, but also to discuss prognosis with a family? The data suggest they are not doing enough of the latter [22]. Future ICU curricula should explicitly include learning objectives around end-of-life conversations, ethical decision-making, and coordination of care, perhaps through simulated family meetings or guided observations in the ICU. Addressing these softer skills will produce more well-rounded physicians who are equipped to handle all facets of critical illness care, not solely the technical aspects.

In conclusion, this scoping review reinforces that ICU-based medical education yields significant educational value and is increasingly recognized as essential. The challenge moving forward is to ensure more universal adoption and to fill the gaps identified. With coordinated effort and continued innovation in teaching methods, medical schools worldwide can better prepare their graduates for the realities of caring for the sickest patients. In summary, this review confirms that ICU education offers crucial benefits, but wider implementation and targeted improvements are needed to maximize its impact and better equip future physicians for critical care challenges.

## Conclusions

ICU-based education offers medical students meaningful exposure to acute care principles and team-based medicine. It is a crucial element of medical training, conferring benefits in knowledge, skills, and confidence that prepare medical students for the demands of residency. Over the past decade, a growing body of evidence shows that early and focused exposure to critical care, whether through dedicated ICU rotations, simulation programs, or integrated curricula, leads to improved competence in recognizing and managing critically ill patients. While existing literature supports the value of ICU experiences, the lack of standardization and long-term outcome data limits generalizability. Effective ICU education employs a blend of bedside teaching, hands-on simulation, case-based learning, and sometimes virtual or remote learning tools to overcome scheduling and resource barriers. These multimodal strategies engage learners and solidify both technical and non-technical skills needed in the intensive care setting.

Critical care education plays an increasingly important role in preparing future physicians to meet the demands of modern clinical practice. Despite demonstrated benefits and growing adoption, ICU training for medical students remains inconsistent and is not yet universally integrated into curricula. Many graduates worldwide begin residency without sufficient critical care exposure, highlighting persistent gaps and variability across institutions and regions. Ongoing efforts are essential to ensure that all medical students acquire the necessary skills to provide safe and effective care from the start of their postgraduate training.

In conclusion, expanding and standardizing ICU-based medical education is crucial for developing a confident, competent physician workforce capable of managing medical emergencies. The past decade has seen meaningful advancements and innovations in this field. By addressing existing educational gaps, medical schools can better incorporate critical care experiences into their programs, ultimately enhancing both learner preparedness and the quality of patient care delivered by future clinicians.

## Appendices

**Table 2:** Appendix 1: search strategy

Database	Search strategy
PubMed	"Medical student"[Mesh] OR "medical education*" AND "Intensive Care Unit"[Mesh] OR "ICU" OR "critical care" AND "Education"[Mesh] OR "teaching" OR "curriculum"
Scopus	TITLE-ABS-KEY("medical student*" AND "intensive care" OR "critical care" OR "ICU" AND education OR teaching OR curriculum)
Google Scholar	“medical education ICU” “medical student” “simulation ICU training” “critical care curriculum" "medical education" "intensive care unit" "ICU" "critical care"
Web of Science	TS=("medical student" OR "medical education" AND ("intensive care" OR "ICU") AND (education OR teaching OR curriculum))
